# A Paternal Kingdom

**Published:** 1893-06-03

**Authors:** 


### The Hospital

An Institutional Journal of

Science, Medicine, Nursing, and Philanthropy.

*For Hospitals, Asylums, Infirmaries, Dispensaries, Medical Practitioners, Students, Nurses, and the Charitable Public.*

Vol. XIV.—No. 349.] June 3, 1893.

### A Paternal Kingdom.

The kingdom of London is a noble parent to its sick, whatever it may be to its healthy and its strong. Parenthood is the highest form of kingship; it is greater than kingship, and includes it as the greater does the less. It is more comprehensive, more helpful, more patient, more tender than brotherhood. The perfect ideal of a perfect state, in the present or in future worlds, is parenthood and childhood. The kingdom of London is not able, may not indeed be willing, to be a parent to every one of her needy and unhappy children; but to the helpless sick she is able and willing and resolute to be a parent.

Where is the proof of this noble intention; where is the proof of the true paternal capacity? For in an ideal parent we do not demand affection merely, but justice, wisdom, strength. We look for the proof, not in the region of logic nor even of poetic imagination: we look for it in homes provided, in necessities met: we look East, West, North, and South: and wherever in this great, wide, and almost measureless London we look, we see the proofs and signs of wise, strong, and capable fatherhood for the sick.

Socialists and other people of eager and restless minds ought to give much consideration to such established things as these. The facts we are about to lay before readers have several kinds of significance attaching to them. They have what may be called their primal and natural significance as mere facts; their significance as works of benevolence and goodness; and their significance as indicators of British and human progress. There are good reasons why all these kinds of significance should be perceived, and should have due attention at the present time; and we desire to press at least these three kinds of significance upon clerical, medical, and general readers.

East, West, North, and South; in whichever direction we look we find that the poor sick are under the most noble and the most capable paternal care. It is a great thing to say: it is an occasion for the most heartfelt mutual congratulation. In the East, although the paternal resources are strained to the utmost, the necessities of the helpless sick are still met. Let us take last year as a sample. In the district included under Stratford and the East-end £119,572 were spent on the sick alone. It is a vast sum to be spent in one district in a single year, and represents a capital of over two and a-half millions of money. For such a sum, representing such a capital, a prodigious amount of doctoring, nursing, lodging, and feeding of the sick ought to be done; and as a matter of fact an amount of work which is quite unrealisable and almost incredible is done. The figures which represent the money spent are very striking; but they seem to us as nothing compared with those which represent the work done. Take first the in-patients and then the out-patients, and then consider them separately. The in-patients in the Stratford and East-end district numbered 15,132 last year, and the out-patients 216,351. But these figures are the numbers of armies. Even the in patients represent a larger army than Queen Elizabeth ever possessed for the defence of her empire; and the out-patients in this one district of London alone are almost half as many in number as the prodigious army of the German Empire at the present moment, and much more numerous than all the efficient British land forces put together. This for one district of London alone. When one considers the vast numbers of people in East London who were housed, fed, nursed, and medically treated last year, one is profoundly astonished that all this scientific, benevolent, and really wonderful work could have been done for £119,572.

But the work done in the East End, and the money spent there, represent considerably less than a fifth of the money spent and the work done in the Metropolis in all its parts. We do not wish to weary readers by a mere array of undigested figures; but for the sake of those who live in the different districts we will indicate very briefly the relative shares of each district in the noble paternal benefits we are now considering, and then we will as briefly indicate the summary of the whole. For the purposes of the Hospital Sunday Fund Greater London is divided into seven districts, and a table issued by the Fund, which we publish on another page, sets forth in detail all the various hospitals and dispensaries in each district, their capacity, their incomes, their expenditure, and the actual work done in each for each year. This tabular statement is sent out broadcast to the clergy, and constitutes an arcana of facts and figures from which sermons of the most pathetic and impressive eloquence may be dug, as from a mine, by those who have eyes to see and hearts to feel.

The seven districts into which London is divided are as follows: Newington and the South, which expends annually £90,896, and treats 14,126 in-patients, and 166,228 out-patients; the City and East Central district, with an expenditure of £54,030, an in-patient roll of 6,899, and an out-patient army of 142,773; Westminster district, expenditure £89,588, in-patients 10,465, out-patients 169,767; St. Marylebone and West Central district, expenditure £108,085, in-patients 10,182, out-patients 179,262; Kensington and West district, expenditure £148,999, in-patients 14,180, out-patients 186,321; Islington and North-West district, expenditure £66,254, in-patients 7,008, out-patients 133,417; and the Stratford and East End district, which, as we have already stated, spends £119,572 annually, and has in-patients numbering 15,132, and 216,351 out-patients. The summary of all this shows that the 136 hospitals and dispensaries of London treated last year 78,172 in-patients, and 1,194,119 out-patients, at a total cost of £677,724 in round figures.

Mr. Stead believes in the lime-light and the screen, and so do we. We believe in every method which makes people see, and understand, and feel. We do not wish them always to see the unhappy and feel the dolorous. Hospitals are not unhappy places; the sight of them and their work is not dolorous. Our chief object in the article we are now writing is to make the supporters of hospitals see and realise the splendid work they have done, and to make them proud of their work and pleased with themselves and their subordinates. But a second and not an unimportant object is to make adverse critics and foolish faddists also see what solid work, standing on solid ground, keeping tens of thousands of useful people alive, and restoring hundreds of thousands of threatened homes to happiness and comfort—what noble work, we say, is done by people who air no fads, who flatter themselves with no cunning theories, but who love plain duty and onest good-will, and who work and do not talk and orate and fill the air with sounding and senseless platitudes.

The hospitals have played a considerable part in the past, but they have a really great part to play in the future, or we are much mistaken. They are the homes of physiology, and therefore of reality; they are the homes of pain and sorrow, and therefore of reality; they are the homes of painstaking science and work, and therefore of reality; they are the homes of manifest, indubitable, life restoring, and joyful success, and therefore of reality. Reality, actuality, solidity, are the notes which must be sounded out through all the ranges of our world-wide empire during the present and the expanding future if our empire is to stand, and to grow and to be great. The faddists will ruin us and our noble empire if we cannot fix ourselves down upon solid realities. The hospitals constitute the most striking object lesson of reality of which we have any knowledge. Whilst science lives and thrives in them luxuriantly, vapid and rootless faddism withers and dies. As object lessons in reality and actuality we will intelligently conserve them, as well as for the beautiful and noble work they do for the poor and the sick.

A word to the practical: and, happily for the hospitals, the Englishman loves the practical word almost better than the visionary and the ideal. We spent last year upon our Metropolitan hospitals £677,724, but we only received £505,843. A sum in subtraction shows a deficit of £171,881 on the year's work. No financial genius is required to tell us what this means. "Income £20, expenditure £19 19s. 6d., happiness: Income £20, expenditure £20 0s. 6d., misery," said Mr. Micawber. It is plain truth The position is here in a nutshell: More money, or closed hospital wards; more money, or London can no longer be a parent to its sick; more money, or the flag of a victorious and glorious benevolence must fly less proudly in the breeze. We refuse to contemplate such a possibility; we will insist upon believing better things until facts make faith impossible. Religion has done more for the sick and the poor than all the other forces of humanity put together. The decay of great deeds announces the decay of faith, and the withering of love. May God avert from us such a decay and such a proclamation.

